# Itraconazole-associated purpuric drug eruption: a rare adverse effect of a commonly prescribed drug

**DOI:** 10.1093/skinhd/vzaf006

**Published:** 2025-03-21

**Authors:** Mahesh Mathur, Sandhya Regmi, Sumit Paudel, Supriya Paudel, Nabita Bhattarai, Sambidha Karki

**Affiliations:** Department of Dermatology, College of Medical Sciences Teaching Hospital, Bharatpur, Nepal; Department of Dermatology, College of Medical Sciences Teaching Hospital, Bharatpur, Nepal; Department of Dermatology, College of Medical Sciences Teaching Hospital, Bharatpur, Nepal; Department of Dermatology, College of Medical Sciences Teaching Hospital, Bharatpur, Nepal; Department of Dermatology, College of Medical Sciences Teaching Hospital, Bharatpur, Nepal; Department of Dermatology, College of Medical Sciences Teaching Hospital, Bharatpur, Nepal

## Abstract

Purpuric drug eruption (PDE) is a rare drug reaction characterized by purpuric macules, papules and confluent plaques predominantly on the lower extremities. The drugs reported to induce PDE are epidermal growth factor receptor inhibitors, ketoconazole, acetylsalicylic acid, penicillin, sulfonamides, indomethacin, lenalidomide, linezolid and vancomycin. Drug-induced thrombocytopenia, platelet dysfunction and direct toxic effects of the drug on the capillary wall leading to increased capillary fragility are the proposed aetiologies. There is only a single report of itraconazole-induced PDE in the literature to date. We herein present a case of 57-year-old woman with PDE due to itraconazole.

## Teaser

Purpuric drug eruption (PDE) is a rare drug reaction characterized by purpuric macules, papules and confluent plaques predominantly on the lower extremities. Drug-induced thrombocytopenia, platelet dysfunction and direct toxic effects of the drug on the capillary wall leading to increased capillary fragility are the proposed aetiologies. There is only a single report of itraconazole-induced purpuric drug eruption in the literature to date. We hereby present a case of 57-year-old female with PDE due to itraconazole.

**What is already known about this topic?**

- Purpuric drug eruption (PDE) is a rare drug reaction characterized by purpuric macules, papules and confluent plaques predominantly on the lower extremities.

**What does this study add?**

- PDE due to itraconazole is extremely rare, so this case is being reported to highlight an uncommon rash induced by a commonly prescribed drug, which will aid in early diagnosis and management.

Purpuric drug eruption (PDE) is a rare drug reaction characterized by purpuric macules, papules and confluent plaques predominantly on the lower extremities.^1^ Itraconazole, despite being one of the most common systemic antifungals used, rarely causes cutaneous reactions.^2^ There is only a single report of itraconazole-induced PDE in the literature to date.^3^ We hereby report a case of PDE associated with itraconazole due to its rarity.

## Case report

**Figure 1:**
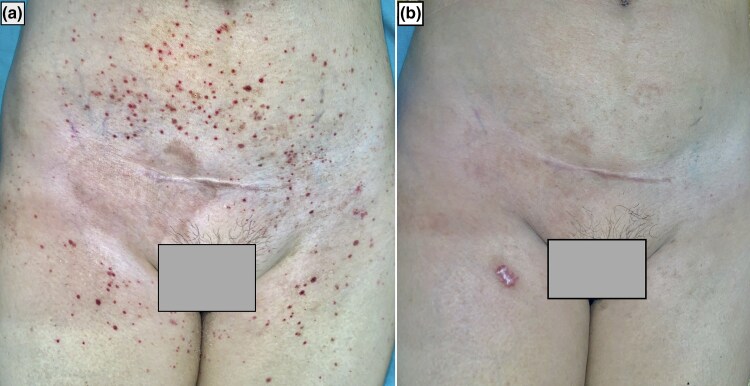
(a) Multiple petechiae and purpura present over lower abdomen, groin and bilateral thighs; (b) on follow-up after 10 days, the rash had regressed completely leaving few hyperpigmented macules.

A 57-year-old woman presented with multiple, asymptomatic petechial and purpuric macules over her bilateral axilla, lower abdomen, groin and bilateral thigh for 4 days (Figure 1a). She denied any systemic symptoms, and there was no history of similar cutaneous lesions.

The skin lesions developed 3 days after intake of oral itraconazole 100 mg twice daily for tinea cruris and gradually progressed as she continued the medication for an additional 4 days. Mucosae examination revealed normal findings.

**Figure 2:**
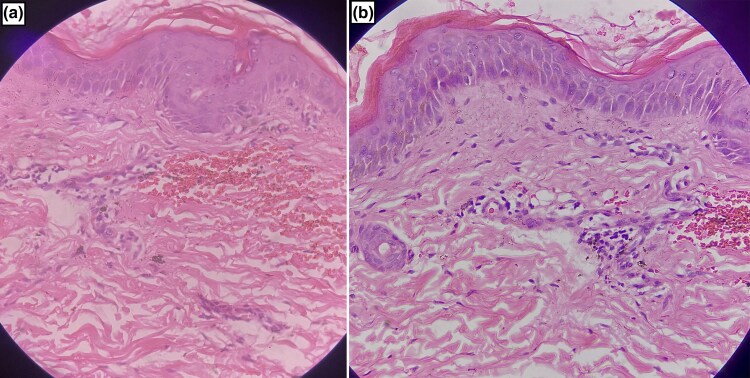
(a, b) Haematoxylin and eosin stain (40×) revealed mild superficial perivascular lymphocytic infiltrates and extravasated red blood cells.

Routine blood investigations performed were normal except for eosinophilia, which was 12%. Additional screening for hepatitis C virus, HIV, hepatitis B virus and antinuclear antibodies were all negative. Coagulation profile and erythrocyte sedimentation rate were within normal limits. Skin biopsy from the right thigh revealed mild perivascular and superficial lymphocytic infiltrates and extravasated red blood cells (Figure 2a, 2b). A separate biopsy for direct immunofluorescence was not taken in our case, as there were no lesions <24 h old. A probable adverse drug reaction to itraconazole was suspected, as the Naranjo adverse drug reaction probability scale was 6. Similarly, the reaction was graded probable as per the World Health Organization–Uppsala Monitoring Centre causality assessment scale. The patient denied consent for an oral rechallenge test. She was counselled regarding the probable drug reaction to itraconazole and advised to avoid triggering medication. As she was asymptomatic, she was only prescribed topical emollients. The skin lesions regressed, leaving few hyperpigmented macules on the 10-day ­follow-up visit (Figure 1b).

## Discussion

PDE is relatively rare and accounts for approximately 1.2% of drug eruptions.^4^ The drugs reported to induce PDE are epidermal growth factor receptor inhibitors, ketoconazole, acetylsalicylic acid, penicillin, sulfonamides, indomethacin, lenalidomide, linezolid and vancomycin.^1,3,5^ Drug-induced thrombocytopenia, platelet dysfunction and direct toxic effects of the drug on the capillary wall leading to increased capillary fragility are the proposed aetiologies.^4^ As the blood investigations were unremarkable except for eosinophilia, a direct toxic effect of itraconazole on the capillaries might be the cause for the PDE seen in our patient.

Itraconazole, a triazole antifungal agent, inhibits fungal cytochrome P450-dependent enzyme, which disrupts ergosterol synthesis in the fungal cell membrane.^6^ Cutaneous adverse drug reactions have been described with itraconazole in 2% of cases and include maculopapular drug eruption, urticaria, angioedema, Stevens–Johnson syndrome/toxic epidermal necrolysis, acute generalized exanthematous pustulosis, vasculitis, symmetrical drug-­related intertriginous and flexural exanthema, and fixed drug eruption.^2,6,7^ PDE induced by itraconazole is extremely rare, and to the best of our knowledge only one case has been reported in the literature so far.^3^ Differential diagnoses include maculopapular exanthem and leucocytoclastic vasculitis, which were excluded by clinical presentation and histopathology.

PDE can be diagnosed on clinical grounds; however, skin biopsy, drug lymphocyte stimulation test and systemic rechallenge help in diagnostic confirmation.^5^ Histopathologically, PDE is characterized by vacuolar interface dermatitis, a sparse superficial perivascular lymphoid cell infiltrate with rare eosinophils and extravasated red blood cells, as described in our patient.^3,4^ Treatment is mainly with topical and systemic corticosteroids and antihistamines; avoidance of the offending drug is of paramount importance.^3,4^

Awareness of unusual drug reactions is crucial, as the association between skin eruptions and drug exposure can often be overlooked or misdiagnosed. PDE due to itraconazole is extremely rare, so this case is being reported to highlight an uncommon rash induced by a commonly prescribed drug, which will aid in early diagnosis and ­management.

## Data Availability

The data underlying this article will be shared on reasonable request to the corresponding author.
